# Intraspecific Colour Variation among Lizards in Distinct Island Environments Enhances Local Camouflage

**DOI:** 10.1371/journal.pone.0135241

**Published:** 2015-09-15

**Authors:** Kate L. A. Marshall, Kate E. Philpot, Isabel Damas-Moreira, Martin Stevens

**Affiliations:** 1 Behavioural Ecology Group, Department of Zoology, University of Cambridge, Cambridge, United Kingdom; 2 Centre for Ecology and Conservation, College of Life and Environmental Sciences, University of Exeter, Penryn Campus, Penryn, Cornwall, United Kingdom; 3 CIBIO Research Centre in Biodiversity and Genetic Resources, InBIO, Universidade do Porto, Campus Agrário de Vairão, Rua Padre Armando Quintas, Vairão, Vila do Conde, Portugal; University of Sussex, UNITED KINGDOM

## Abstract

Within-species colour variation is widespread among animals. Understanding how this arises can elucidate evolutionary mechanisms, such as those underlying reproductive isolation and speciation. Here, we investigated whether five island populations of Aegean wall lizards (*Podarcis erhardii*) have more effective camouflage against their own (local) island substrates than against other (non-local) island substrates to avian predators, and whether this was linked to island differences in substrate appearance. We also investigated whether degree of local substrate matching varied among island populations and between sexes. In most populations, both sexes were better matched against local backgrounds than against non-local backgrounds, particularly in terms of luminance (perceived lightness), which usually occurred when local and non-local backgrounds were different in appearance. This was found even between island populations that historically had a land connection and in populations that have been isolated relatively recently, suggesting that isolation in these distinct island environments has been sufficient to cause enhanced local background matching, sometimes on a rapid evolutionary time-scale. However, heightened local matching was poorer in populations inhabiting more variable and unstable environments with a prolonged history of volcanic activity. Overall, these results show that lizard coloration is tuned to provide camouflage in local environments, either due to genetic adaptation or changes during development. Yet, the occurrence and extent of selection for local matching may depend on specific conditions associated with local ecology and biogeographic history. These results emphasize how anti-predator adaptations to different environments can drive divergence within a species, which may contribute to reproductive isolation among populations and lead to ecological speciation.

## Introduction

Intraspecific variation of colour patterns is a long studied phenomenon in animals, and has profoundly contributed to our understanding of evolutionary processes. Classic experiments have shown that natural selection can drive variation in coloration among populations to optimize survival against predators in varying local environments ([1–3]). It is now understood that adaptation to local environments can cause reproductive isolation between divergent populations and potentially lead to ecological speciation (reviewed in [4–6]).

Various studies on mice and lizards have shown that divergent populations undergo selection to better match local backgrounds for camouflage, and that this involves mutations of key genes and is influenced by levels of gene flow (e.g. [7–11]). However, until recently, relatively few studies have based their conclusions on the visual systems of potential predators, such as hunting birds (but see [12, 13]). As the avian predator visual system and perception of background matching camouflage differs from that of humans (for example, birds can see ultraviolet light and probably a greater range of colours; [14, 15]), it is important to determine whether predators perceive divergence in camouflage among local environments (e.g. [16, 17]). As local adaptation for camouflage should involve selection for coloration that matches associated backgrounds, it can be inferred only if local background environments are different. This is because coloration could become variable through other processes, such as genetic drift, even among similar environments. Variation may also arise owing to phenotypic or developmental plasticity, whereby individuals can change colour during their lifetimes (e.g. [18–21]). Consequently, it is important to determine how predator-perceived camouflage and visual backgrounds vary among populations in order to understand local adaptation and its role in intraspecific divergence.

**Fig 1:**
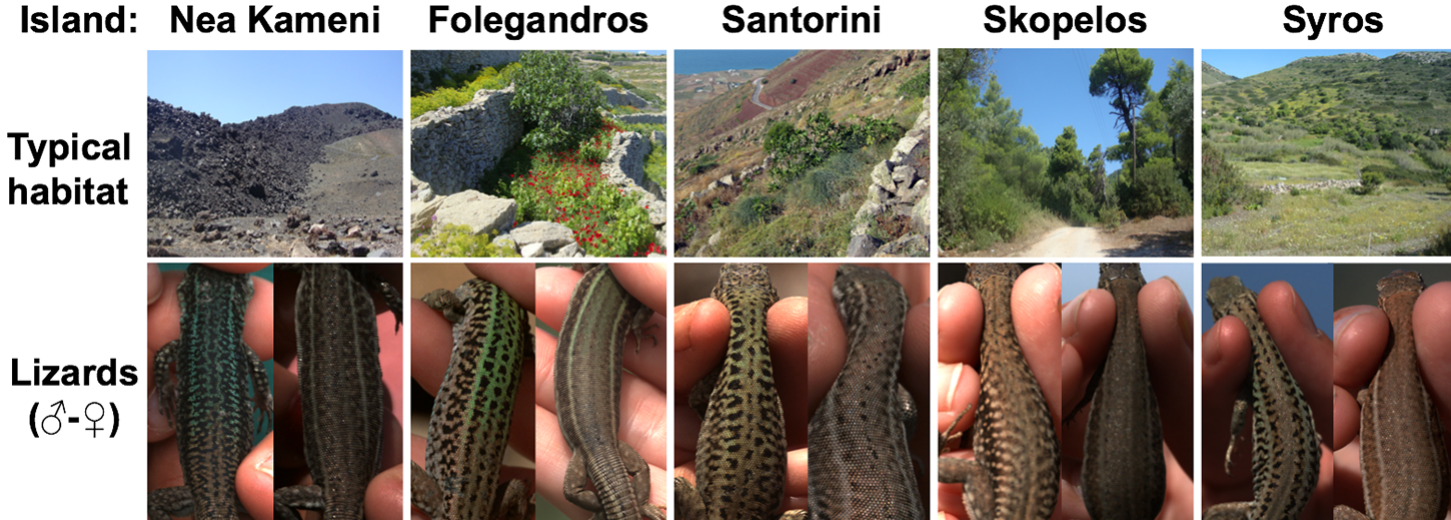
Example images of Aegean wall lizards (*Podarcis erhardii*) and their typical island habitats. Images depict typical natural habitat and dorsal coloration of males (left image) and females (right image) in each focal island population (Nea Kameni, Folegandros, Santorini, Skopelos and Syros). Images were obtained in the field by the authors.

The Aegean wall lizard (*Podarcis erhardii;* [22]) is a valuable model species for this type of study because different island populations exhibit clearly visible colour variation (to humans) among ostensibly distinct island environments (see Fig 1). These island populations have been historically categorised as different subspecies due to their varying appearance ([23]; but see more recent molecular analyses in [24, 25]) and show genetic diversity between isolated islands with no recent common history ([26]). The variable environments of the islands are widely thought to have driven the apparent phenotypic and genetic diversification among the inhabitant *P*. *erhardii* populations (via the equivalent of a sustained bottleneck) dating from their time of separation ([25–28]), although this has not been formally tested.

Studying *P*. *erhardii* in this island system differs from a range of previous studies focusing on the adaptive significance of colour polymorphisms among sympatric lizard populations (e.g. [7, 9, 13, 29]). In particular, examining colour variation between isolated island populations can potentially reveal how distinct ecological factors, as well as time of isolation and evolutionary history (e.g. historical geographical connections), may drive differential local adaptation of camouflage and thus further clarify the selective conditions under which it can occur. Specifically, island populations of *P*. *erhardii* vary in habitat type (e.g. geological characteristics and habitat composition), predation risk (i.e., number of resident avian predators), time of isolation (ranging from >200,000 to 400 years), and potential amounts of gene flow from other island populations depending on historical geographic connections and amounts of boat traffic (see Table 1; [25–28, 30–32]). For example, the Nea Kameni islet has been formed from intermittent volcanic eruptions over the last 400 years ([33]) and inhabitant *P*. *erhardii* rest on homogeneous black lava rock backgrounds, whereas the land bridge island of Syros has been isolated for over 13,000 years and lizards rest on various metamorphic rock backgrounds (Table 1; [27, 34]).

**Table 1:** Differences in the environments and evolutionary histories of the five focal islands inhabited by populations of Aegean wall lizards (*Podarcis erhardii*).

Island and subspecies ^a^	Time of isolation (years)^b^	Historical geographical connections^b^	No. avian predator species^d^	Main habitat type	Main rock type^e^
**Folegandros** *P*.*e*. *naxensis*	11,650	Syros, Santorini	2	Rocky shrubland	Metamorphic (marble, schist)
**Nea Kameni** *P*.*e*. *naxensis*	400 (65) ^c^	None; formed from volcanic eruptions	5	Lava dome	Igneous (black dacite)
**Santorini** *P*.*e*. *naxensis*	> 200,000	Folegandros, Syros	5	Rocky shrubland	Various (e.g. rhyolite, pumice, limestone, schist)
**Skopelos** *P*.*e*. *ruthveni*	5,900	None; separate island group	4	Pine forests, some fields	Sedimentary (dolomites, breccias, limestone)
**Syros** *P*.*e*. *mykonensis*	12,800	Folegandros, Santorini	5	Rocky shrubland	Metamorphic (schist, gneiss, marble)

^a^ Subspecies identified following [23–25].

^b^Estimated date of separation from adjacent landmasses (except Nea Kameni; [27])

^c^The last volcanic eruption in 1950 probably eradicated the Nea Kameni population, so the current population age is approx. 65y [26].

^d^ From [31]. Major avian predator species considered are: *Buteo buteo*, *Buteo rufinus*, *Falco tinnunculus*, *Falco eleonorae*, *Tyto alba*, *Corvus corax* and *Corvus corone*.

^e^ From [34, 35].

A recent study showed that *P*. *erhardii* island populations are tuned to their local island environments to be conspicuous to conspecifics while also being camouflaged against avian predators ([36]). Specifically, *P*. *erhardii* coloration was found to be less perceptible by avian predators than by conspecifics on all three focal islands. Moreover, dorsal regions (upper and lower backs) exposed to aerially hunting birds were relatively more camouflaged against local rock backgrounds compared to less exposed lateral (flank) regions, and this was more evident on some islands compared to others. In addition, on all islands, sexually competing males showed inferior camouflage on their upper backs compared to that of females. Given the apparent environmental variation among the islands, these findings suggest that *P*. *erhardii* have adapted to their local environments to enhance dorsal camouflage against avian predators, and also that degree of local adaptation varies among islands, and between sexes and upper and lower backs. However, this study did not test whether the backgrounds and lizard populations were variable among islands, or whether lizards were more camouflaged against their local island backgrounds compared to that of different islands. Therefore, in the current study we directly test these hypotheses, and also consider how different ecological selection pressures (e.g. predation risk) and evolutionary history (e.g. time of island isolation) has influenced local selection on dorsal camouflage in each population (Table 1).

It is likely that island populations of *P*. *erhardii* have adapted to be camouflaged in their local environments for several reasons. The populations are under considerable though variable threat from avian predators (i.e., raptors and corvids), particularly as they tend to bask on rocks in open environments where they are often visible to hunting birds, as in other lizards (Table 1; [31, 37–39]; pers. observations). Therefore, it is likely that selection has favoured colour matching against local rock backgrounds to reduce detection by avian predators. However, the variable threat from avian predators among the islands may affect degree of local adaptation for camouflage, as shown in past work on other lizards (e.g. [13, 29, 40]). Additionally, various studies have shown that there is relatively little gene flow (i.e., via over-water dispersal) between most island populations ([26–28, 41]), which is similar to other lizards showing phenotypic diversification among recently isolated islands where dispersal across seawater is also extremely rare ([42]). Thus, most of the current *P*. *erhardii* populations are highly likely to be descendants of the original colonists that have adapted to their native environments since they became isolated from neighbouring landmasses. This is substantiated by past work showing genetic adaptation of lizard coloration to environments that have existed for only 6,000 years (e.g. [10, 29]), which is a comparable age to most island environments considered in the current study (Table 1; [27]).

We investigated whether *P*. *erhardii* populations are more camouflaged on their local island than on non-local islands to avian predators, and whether this was associated with lizard and environmental (substrate) differences in appearance between local and non-local islands. We predicted that there would be differences in coloration between *P*. *erhardii* island populations and between their rock background environments, and that lizards would show better camouflage against local backgrounds than against non-local backgrounds to avian predators. We also predicted that there would be variation in degree of camouflage among islands with different evolutionary histories and environments, and between sexes and body regions.

## Materials and Methods

### Study species and sites

The Aegean wall lizard (*Podarcis erhardii*) is a diurnal, small lacertid distributed across most of the South Balkans and widespread throughout many Aegean islands where it is found in all ecosystems ([43, 44]). We conducted field research during the activity season (April-July; [45]) between 2012–2014 on five Aegean islands with three different inhabitant subspecies (classified based on appearance; [23]): *P*.*e*. *naxensis* (Folegandros [36°37’ N, 24°54’ E], Santorini [36°25’ N, 25°26’ E] and the neighbouring islet Nea Kameni [36°24’ N, 25°24’ E]; *P*.*e*. *mykonensis* (Syros [37°27’ N, 24°54’ E]); and *P*.*e*. *ruthveni* (Skopelos [39°7’ N, 23°43’ E]). We chose to sample lizards from these islands because they have ostensibly varying coloration and environments, substantial but variable risk from avian predators, and different evolutionary histories (see Fig 1; Table 1).

### Ethics statement

*P*. *erhardii* is listed as a species of ‘least concern’ under the IUCN Red List classification, so it is not an endangered or protected species ([46]). We conducted field research with permission from the Greek Ministry of Environment (permit numbers: 166648/356 and 107222/707). These permits were granted for all study sites, and all land used for fieldwork was publicly accessible. The permits also gave us permission to sample lizard coloration using all methods as described. Sampling methods were non-invasive, involving photography of lizards in situ. However, in one study site (Nea Kameni islet) lizards were captured for photography, because this islet has a low population density and we had restricted access with a limited amount of time to obtain an appropriate sample size. Lizards were caught using a noose consisting of a slipknot attached to a telescopic pole, which is widely used by herpetologists as a non-harmful capture method (e.g. [47]). Nonetheless, we endeavoured to release the lizard from the noose as quickly as possible after capture, which was usually within five seconds and never more than 10 seconds. All researchers had prior experience using this capture method and with handling lizards. After capture, lizards were immediately photographed, marked with a small amount of non-toxic (water-based), inconspicuous (dark green) paint (Montana, Heidelberg, Germany) on the vertical surface of their hind leg so as not to alter dorsal camouflage, and then released at the original (marked) point of capture. During capture, we ensured that we handled lizards in a way that minimized the likelihood of tail autotomy and aimed to release them as soon as possible, which was typically within five minutes. Lizards were never harmed and all showed normal behaviour after release. We captured the minimum number of lizards necessary to rigorously test our hypotheses (*N* = 32; 19 males, 13 females). All protocols conformed to the policies and requirements of the ethics committee of our institution.

### *In situ* photography

We used digital imaging to sample lizards and their corresponding background coloration, because it has various advantages over alternative spectrometry methods (see [36, 48]). As in ([36]), during in situ photography we took images of stationary lizards and their corresponding natural backgrounds with a Fujifilm IS Pro ultraviolet (UV)-sensitive digital camera with a quartz CoastalOpt UV lens (Coastal Optical Systems), fitted with a UV and infrared (IR) blocking filter for photographs in the human-visible spectrum (Baader UV/IR Cut filter; transmitting between 400 and 700 nm), and with a UV pass filter (Baader U filter; transmitting between 300 and 400nm) for UV images. After the photographed lizard had fled, we took human-visible and UV images of a Spectralon^TM^ grey reflectance standard (Labsphere, Congleton, UK), which reflects light equally at 40 per cent between 300 and 750 nm. Following this ‘sequential method’ as used in past work ([49, 50]), images of the standard were taken at the same distance, with the same camera settings, and in the same location and light conditions, as the photographed lizard. This enabled us to undertake the required standardization for ambient light conditions (i.e. image linearization and RGB-equalization; see below) (see [48]). Light conditions rarely changed in the typical 2–3 minutes between photographing the lizard and the standard. However, on the few occasions when this did occur (e.g. clouds passed over the sun), we re-photographed the standard once the lighting had returned to normal. We endeavoured to photograph most lizards (and hence standards) in full sunlight. In the few cases where shadows partly obscured the image, our selections avoided shadowed areas and only measured parts of the lizards, backgrounds and standards that were in sunlight.

We recorded photographed lizards’ locations using a Garmin eTrex GPS device (Schauffhausen, Switzerland) and marked it with coloured tape to indicate sex and life-stage ([43]). We confirmed these estimations were 99% reliable by comparing estimated (from photographs) and observed sex and life-stages from captured lizards (see [36]). We avoided pseudoreplication by never repeating photography of a lizard of the same sex within the same home range (i.e. within 10 m) (see [36, 51]).

On the Nea Kameni islet we captured lizards for photography. Capture location was marked with coloured tape for subsequent release. To minimise stress and to avoid any colour fading through decreases in body temperature, which can sometimes arise during long-lasting stressful situations in lizards (e.g. [52, 53]), lizards were photographed within five minutes after capture. The grey reflectance standard was photographed and lizards were marked as described previously. Observations and other mark re-capture studies showed that marks lasted for at least three weeks (K. Marshall and I. Damas-Moreira, unpublished data). After allowing the paint to dry, lizards were released at the marked capture location. Capture attempts were not made within 10m of the same location again. To ensure that lizards would be subsequently compared to their natural corresponding backgrounds, each (marked) background that each lizard had been resting on at the point of capture was photographed with the grey standard in the image.

### Image analysis and visual modelling

We followed methods previously described in ([36]). Human-visible and UV images of lizards and their backgrounds were linearized with respect to light intensity and transformed to reflectance (RGB-equalized; see [48]). Any images that were overexposed and/or could not be RGB-equalized were discarded from the analysis. We then used a mapping process based on the spectral sensitivity of our camera’s sensors (derived prior to photography; see [36]) to convert the images to correspond to avian predicted photon catch cone values ([48]). This is highly accurate compared to spectrometry-derived estimates of animal photon catch values (see [54–56]). We converted the aligned images from camera colour space to the relative photon catches of an avian predator’s longwave (LW), mediumwave (MW), shortwave (SW) and UV sensitive cone photoreceptors using the spectral sensitivity of a peafowl (*Pavo cristatus*; [14]). We also mapped the images to peafowl double cone photoreceptors ([57]) to represent achromatic (luminance) avian perception ([58, 59]). The peafowl visual system is often used as a representative of the violet-sensitive (VS) class of colour vision in birds ([60, 61]). The VS-system is typical of the predatory birds that hunt *Podarcis* lizards and other lacertids in Europe (i.e. raptors and corvids; [31, 37, 62]), which has recently been supported by molecular studies tracing the evolution of ultraviolet vision in birds ([63]). Although some birds with ultraviolet-sensitive (UVS) vision might also prey on *P*. *erhardii* (e.g. gulls and some Turdidae (thrush) species that are present in our study sites; [63–66]), we considered VS-sensitive raptors and corvids as the more important avian predators of adult lizards in Europe ([31, 37, 62, 63, 67]). Indeed, we often observed hooded crows (*Corvus cornix*) and a variety of raptors (e.g. buzzards, *Buteo buteo*; common kestrels, *Falco tinnunculus;* Eleonora’s falcon, *Falco eleonorae*) hunting in our study sites, while gulls and thrushes were rarely seen. Calibrations were performed in MATLAB v. R2011b (The MathWorks, Inc., MA, USA) using self-written programs. Both calibrations were restricted to the 300–700 nm range, which encompasses most of the visual spectrum of diurnal birds ([61]).

LW, MW, SW and UV photon catches of each lizard and their corresponding background (i.e., the background we photographed the lizard resting on) were extracted from the calibrated images in ImageJ using the selection tool. Selection of a particular area in the image generated an average photon catch value for that patch. Background selections were limited to rock backgrounds because *Podarcis* lizards most frequently bask on rocks (given their good thermal quality) where they are potentially visible to aerially hunting predators in open environments (e.g. [38, 39, 68]; pers. observations). We randomly selected areas of rock that were adjacent to the photographed lizard, so that the selection touched but did not overlap it, and avoided areas of lichen and moss. Lizards sometimes rested on rock backgrounds that consisted of different patches of colour patterns, although we could usually identify the predominant (largest) patch from which we made our background selection. However, in some images we could not identify a predominant background patch. In these cases, we made up to three selections of different patches and averaged the photon catches across patches as a representative substrate colour.

Lizard selections were made from two dorsal body regions (posterior lower and anterior upper backs). The average photon catch value extracted by these selections included dorsal patterning, which is particularly extensive in males (see Fig 1). We assumed that this would depict how lizards appear to birds hunting from a distance, especially given that the lizards are small (i.e. ≈7cm snout-to-vent length; [43]). However, we acknowledge that at close range patterning might be more distinguishable by birds and serve another function (e.g. disruptive camouflage; [69]). Separate selections of lower and upper backs were taken due to observed colour differences between these regions, and selection of these body regions was standardized across all images. Specifically, upper back selections were taken under the base of the head and lower backs selections were taken above the base of the tail (see [36]). A total of 295 adult lizards and their corresponding rock backgrounds were sampled from the five focal island populations (Folegandros = 100; 52 males, 48 females, Syros = 49; 34 males, 15 females; Santorini = 58; 34 males, 24 females; Skopelos = 56; 29 males, 27 females; and Nea Kameni = 32; 19 males, 13 females).

### Island differences in lizards and backgrounds

We first aimed to ascertain any differences in colour and luminance among the island lizard populations and their rock background environments. We calculated a variety of metrics to use in our analysis. *Saturation* (the amount of a certain colour compared to white light) refers to the Euclidian distance a colour patch is in a tetrahedral colour space from the achromatic (grey) point, with larger values representing more saturated colours (see [16]). We also quantified the type of colour (*hue*). This was based on a ratio of the different colour channels present (i.e., LW, MW, SW and UV) to represent relative photoreceptor stimulation by the colour patch spanning different parts of the light spectrum and receptor types ([55, 70–72]). This followed an approach developed in past work, which aims to quantify colour so that it broadly reflects how colour is encoded by an animal’s visual system (see [56, 71, 72]). Specifically, we used a ratio based on the concept that colour is encoded using opponent (antagonistic) colour channels or neural pathways, such as the ‘red-green’ and ‘blue-yellow’ pathways in trichromatic primates ([59, 73–75]). Since it is unknown which specific opponent colour channels birds have, we identified biologically relevant putative colour channels by performing a principle component analysis (PCA) on a covariance matrix of the standardized cone photon catches derived from our avian visual model ([71]). This enabled us to determine one or two colour channels that best described the variation in colours existing among lizards ([56, 72]). Two PCs were extracted and together explained almost all of the variance in lizard and background coloration (lower backs = 95.2%; upper backs = 93.6%; backgrounds = 97.8%). PC1 showed highest eigenvalue loadings on the LW and SW channels, and PC2 showed highest loadings on the MW and UV channels. Therefore, we used the following standardized ratio to identify perceived differences in the identified opponent colour channels ((LW+SW)-(UV+MW)/UV+SW+MW+LW); see [69, 71]). Consequently, this ratio would reflect differences between perceived ‘purple’ and ‘green-UV’ coloration. Finally, for a measure of *luminance* (i.e. perceived lightness), we used standardized double cone values derived from the avian visual model ([57–59]).

### Degree of island differences in lizards and backgrounds

In addition to calculating the *hue*, *saturation* and *luminance* metrics separately, we sought more insight into the divergence between islands by determining the extent of the perceived differences in lizards and their rock backgrounds, and to understand perceptual differences in colour accounting for both *hue* and *saturation* together. To do this, we measured the degree of contrast between each island population (i.e., lizards vs. lizards) to identify how similar lizards were to members of their local population and to members of non-local populations. We also measured the degree of contrast between the measured backgrounds (i.e., backgrounds vs. backgrounds) to identify how similar rock backgrounds were to those of the local island and to those of non-local islands. We quantified degree of colour (chromatic) contrast according to the widely used log form of a receptor noise model ([76]), which predicts visual discrimination abilities in observers. We also quantified luminance contrasts using a version of the model based on achromatic differences using peafowl double cones ([77]). To account for receptor noise, we used a Weber fraction value of 0.05 for the most frequent cone type based on data in other vertebrates ([76, 78]). We used relative proportions of cone types in the peafowl retina to calculate avian predator-perceived chromatic contrast (LW = 0.92, MW = 1.00, SW = 0.81, UV = 0.54; [14]). The degree of chromatic and achromatic contrast generated from these models is expressed as a measure of contrast called “just-noticeable-difference” (JND), which predicts discrimination behaviour in observers. Generally, JND values increasing above 3.00 indicate increasingly improved discrimination ([77]).

We compared each lizard with every lizard belonging to all island populations, including to its local population and to all four non-local populations. We performed the same comparisons with the backgrounds to determine whether differences in visual backgrounds were large enough to be perceptibly different to avian predators, and thus cause changes in lizard appearance to match them on each island. To control for sexual dichromatism in lizards’ dorsal coloration and variation in coloration between upper and lower backs ([36]), lizards were compared with only members of the same sex and only equivalent body regions were compared. After obtaining JNDs for all comparisons, we calculated the average JND (i.e., degree of contrast) for each lizard and each background to its local island and to each of the four non-local islands.

### Local adaptation of camouflage

Lastly, we determined to what extent the lizard populations matched different (local and non-local) rock backgrounds for background-matching camouflage. To ascertain any differences in camouflage among islands, we used the receptor noise model described above ([76]) to compare each lizard with all local island backgrounds and with all non-local backgrounds from the four different islands. We calculated the average JND for each lizard to ascertain how well they matched local and non-local island backgrounds.

### Predictions and statistical analyses

Normality tests and residuals analysis showed a normal distribution of *hue*, but the *saturation*, *luminance* and JND data were not normally distributed with positively skewed distributions. Therefore, we transformed this data to normality using a logarithmic transformation and used the transformed data in all statistical analyses. However, to illustrate and describe our results, we report raw (back-transformed) JND data in figures. All statistical analyses were conducted in SPSS^®^ (v20).

First, we predicted that coloration and luminance of lizards and their rock backgrounds would differ among islands and be more similar to those on local islands as opposed to those on non-local islands, particularly between islands that have never shared a historical geographical connection and that have different subspecies (see Table 1). We also expected lizards to vary between sexes and body regions due to sexual dichromatism ([36]). To address these predictions, we first conducted a series of general linear models (GLMs) on the colour (*hue* and *saturation*) and *luminance* metrics. To test for differences among island backgrounds, we carried out univariate GLMs with island (Folegandros, Nea Kameni, Santorini, Skopelos and Syros) included as the between-subjects factor. To test for differences in lizard populations, we conducted mixed GLMs with island and sex as between-subjects factors and body region (upper and lower backs) as the within-subjects factor. We also conducted mixed GLMs on the chromatic and achromatic lizards vs. lizards JND data (tests 1 and 2) and backgrounds vs. backgrounds JND data (tests 3 and 4). In tests 1 and 2, island and sex were included as between-subjects factors and body region (upper and lower backs) and ‘compared island’ (i.e., which island the compared lizard belonged to) were included as the within-subjects factors. The same variables were included in tests 3 and 4, with the exception of body region and sex.

Second, we predicted that lizards would match their local backgrounds better than backgrounds on other islands for camouflage against avian predators, and that this would be accompanied by environmental (rock background) differences between islands. In addition, we predicted that this effect would be weaker in populations that have been isolated for relatively shorter evolutionary periods and have ecological characteristics that reduce the need for camouflage, such as lower levels of risk from avian predators (see Table 1). We also predicted that females would show better local adaptation for camouflage compared to sexually competing males, and that lower backs would show enhanced local camouflage compared to sexually dichromatic upper backs. To address these predictions, we conducted two mixed GLMs, one testing chromatic contrast (test 5) and the other analysing achromatic contrast (test 6) against backgrounds (JND). In both GLMs, we included sex and island lizard population as between-subjects factors. The ‘compared island’ backgrounds (i.e. which island background the lizard was compared to) and body region were included as within-subjects factors. In both tests 5 and 6 we specifically looked for a factor interaction between island lizard population and ‘compared island’ background to determine if degree of camouflage (JND) depended on whether lizards were compared with their local island background or with a different (non-local) island background.

In all tests we also report any significant effects of sex and body region on lizard differences between islands and degree of local adaptation of camouflage. We report the size of all effects in ETA^2^ (η^2^), which can be interpreted as the proportion of variance in the dependent variable that is attributable to each effect. To determine the sources of variation in any significant effects, planned pairwise comparisons were conducted by re-running the GLMs with only the factors of interest included that were relevant to our predictions. Factors with non-significant effects were removed from the analysis before re-running the GLM to obtain the best model. The number of comparisons was limited to the number of “spare” experimental degrees of freedom (n-1), because these are more powerful than conservative, multiple unplanned post hoc comparisons ([79]).

## Results

### Island divergence in lizards and backgrounds

As predicted, lizard coloration (*hue* and *saturation*) and *luminance* varied between some but not all island populations, and rock backgrounds were also variable between most islands (see Figure A and Tables A and B in S1 File). Differences between islands were most evident in terms of *luminance* and patterns of variation were generally similar in lizards and backgrounds (see Figure A in S1 File). For example, Nea Kameni lizards were darker than all other populations, and Syros lizards were lighter than all populations except Santorini (see Figure A and Table A in S1 File; *F*_4, 290_ = 28.346, *P* < 0.001, η^2^ = 0.281). Accordingly, Nea Kameni rock backgrounds were also darker than those on all other islands, and Syros rocks were lighter than those on Skopelos and Nea Kameni (see Figure A and Table B in S1 File; *F*_4, 290_ = 37.755, *P* < 0.001, η^2^ = 0.342).

Differences in colour (*hue* and *saturation*) between lizard populations were less evident and usually found in only one sex and mainly in upper backs (Table A in S1 File; island*body region: *F*_4, 285_ = 4.997, *P* = 0.001, η^2^ = 0.066; island*sex: *F*_5, 285_ = 3.316, *P* = 0.006, η^2^ = 0.005). For example, upper back coloration was more saturated in Nea Kameni lizards than in the Syros population (Figure A in S1 File; *F*_1, 79_ = 6.140, *P* = 0.015, η^2^ = 0.072) and differences in perceived purple and UV-green coloration (*hue*) were lower in Nea Kameni males compared with Santorini and Folegandros males (Table A in S1 File; vs. Folegandros: *F*_1, 69_ = 7.978, *P* = 0.006, η^2^ = 0.104; vs. Santorini: *F*_1, 51_ = 6.478, *P* = 0.014, η^2^ = 0.113). A similar trend in variation was found in the rock backgrounds of these populations (Figure A and Table B in S1 File).

### Degree of island differences in lizards and backgrounds

Mauchly’s Test of Sphericity showed that the assumption of sphericity had been violated in the ‘compared island’ factor (test 1: χ^2^_(9)_ = 251.639, *P* < 0.001; test 2: χ^2^_(9)_ = 666.118, *P* < 0.001; test 3: χ^2^_(9)_ = 345.430, *P* < 0.001; test 4: χ^2^_(9)_ = 669.401, *P* < 0.001; test 5: χ^2^_(9)_ = 373.429, *P* < 0.001; test 6: χ^2^_(9)_ = 925.522, *P* < 0.001). Therefore, the Greenhouse-Geisser correction was used when reporting results for this factor.

**Fig 2:**
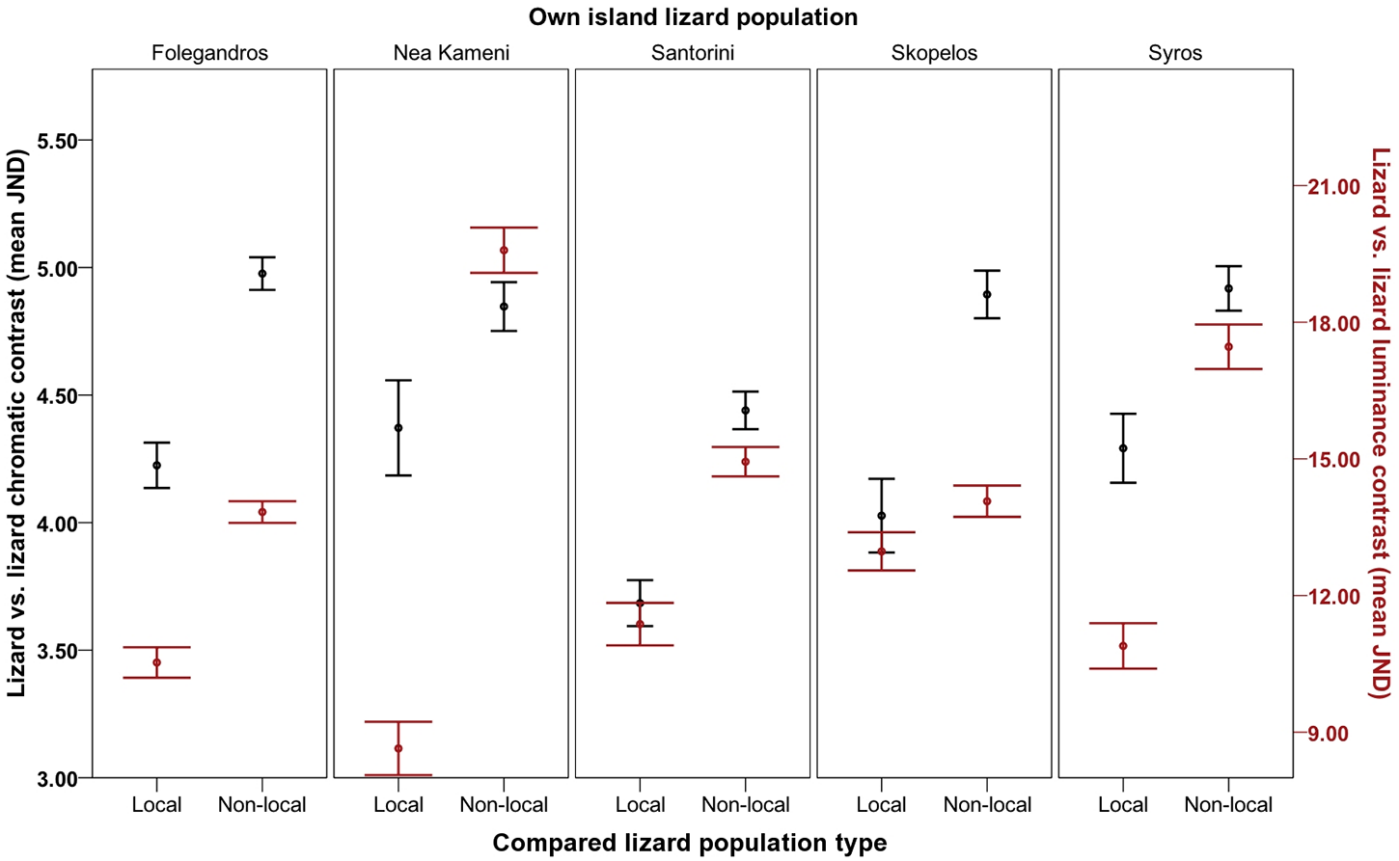
Degree of lizard contrast between island populations. Degree of contrast refers to how different Aegean wall lizards (*Podarcis erhardii*) are to their local island population relative to how different they are to other (non-local) island populations overall (Folegandros, Nea Kameni, Santorini, Skopelos and Syros) in terms of chromatic contrast (left axis; black data points) and luminance contrast (right axis; red data points) (mean JND). JND values increasing >3.00 depict populations that are progressively distinguishable by avian predators. Error bars represent +/- 1 S.E.

As predicted, lizard dorsal regions were more similar to that of members of their own population than to those of other populations (Fig 2). In both chromatic and achromatic avian visual models, this effect varied among islands and depended on which island populations were compared (*chromatic*: *F*_11.690, 832.911_ = 15.476, *P* < 0.001, η2 = 0.178; *achromatic*: *F*_7.760, 552.911_ = 20.849, *P* < 0.001, η2 = 0.226; see supporting information for more details [Table A and Figure A in S2 File). Moreover, this overall effect varied with sex and with body region in both models (*chromatic*: sex, *F*_14.612, 832.911_ = 11.553, *P* < 0.001, η2 = 0.169; body region, *F*_13.404, 955.027_ = 6.608, *P* < 0.001, η2 = 0.085; *achromatic*: sex: *F*_7.760, 552.911_ = 4.205, *P* < 0.001, η2 = 0.056; body region: *F*_11.846, 844.019_ = 8.365, *P* < 0.001, η2 = 0.029). As predicted, in most populations, avian predators perceived upper backs to be more variable than lower backs, and males to be more variable than females, regarding both colour and luminance when compared to other island populations (Table A in S2 File).

**Fig 3:**
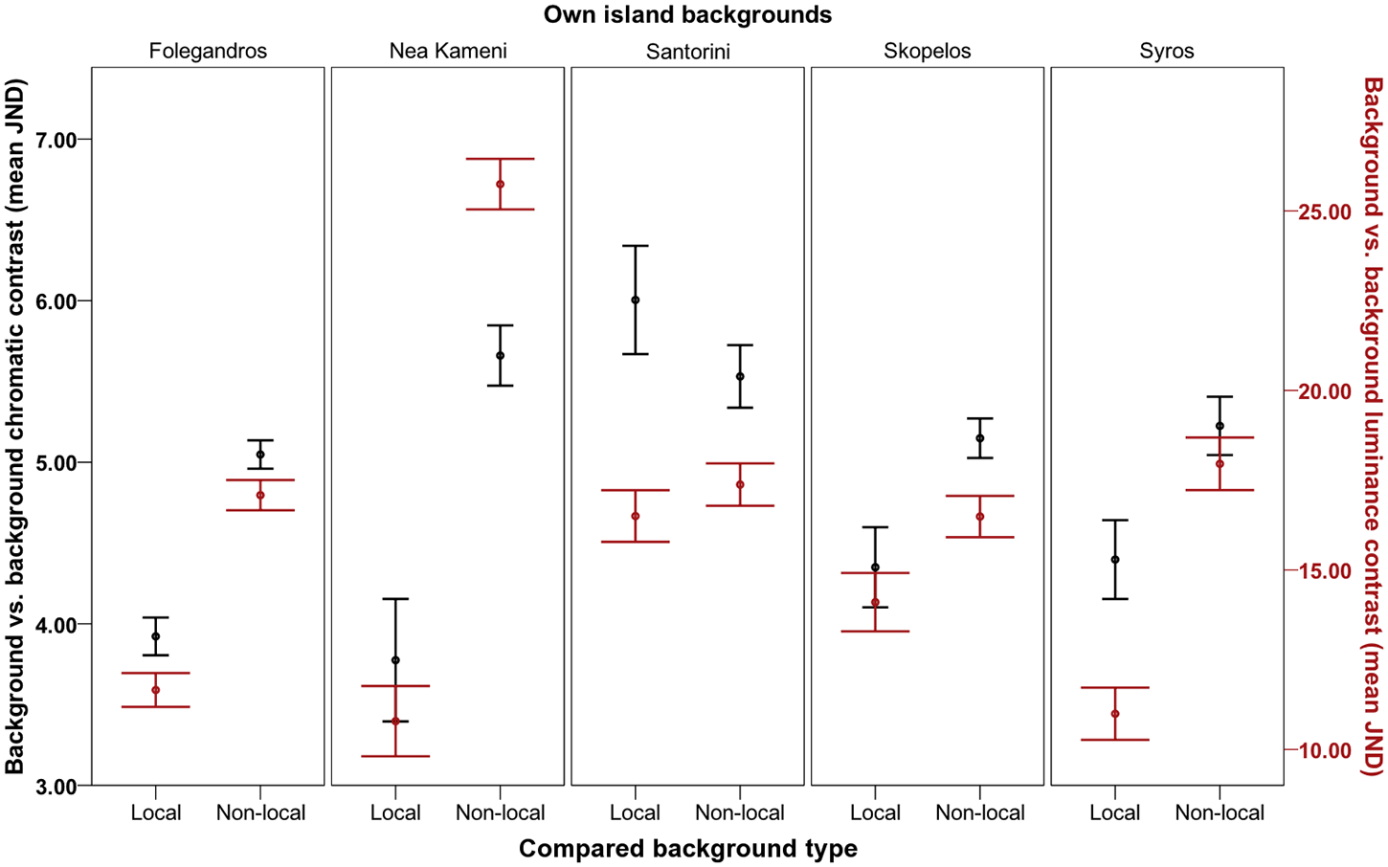
Degree of rock background contrast between islands. Degree of contrast refers to how different Aegean wall lizard (*Podarcis erhardii*) rock backgrounds are to that of their local island population relative to how different they are to the backgrounds of other (non-local) island populations overall (Folegandros, Nea Kameni, Santorini, Skopelos and Syros) in terms of chromatic contrast (left axis; black data points) and luminance contrast (right axis; red data points) (mean JND). JND values increasing >3.00 depict rock backgrounds that are progressively distinguishable by avian predators. Error bars represent +/- 1 S.E.

As in the lizards, rock backgrounds were more similar to those on local islands compared to those on different islands, as predicted (Fig 3). Again, this effect varied among islands and depended on which islands were compared, in both visual models (Table A and Figure B in S2 File; chromatic: *F*_10.075, 730.407_ = 10.164, *P* < 0.001, η2 = 0.123; achromatic: *F*_7.055, 511.458_ = 30.001, *P* < 0.001, η2 = 0.293).

### Local adaptation of camouflage

**Fig 4:**
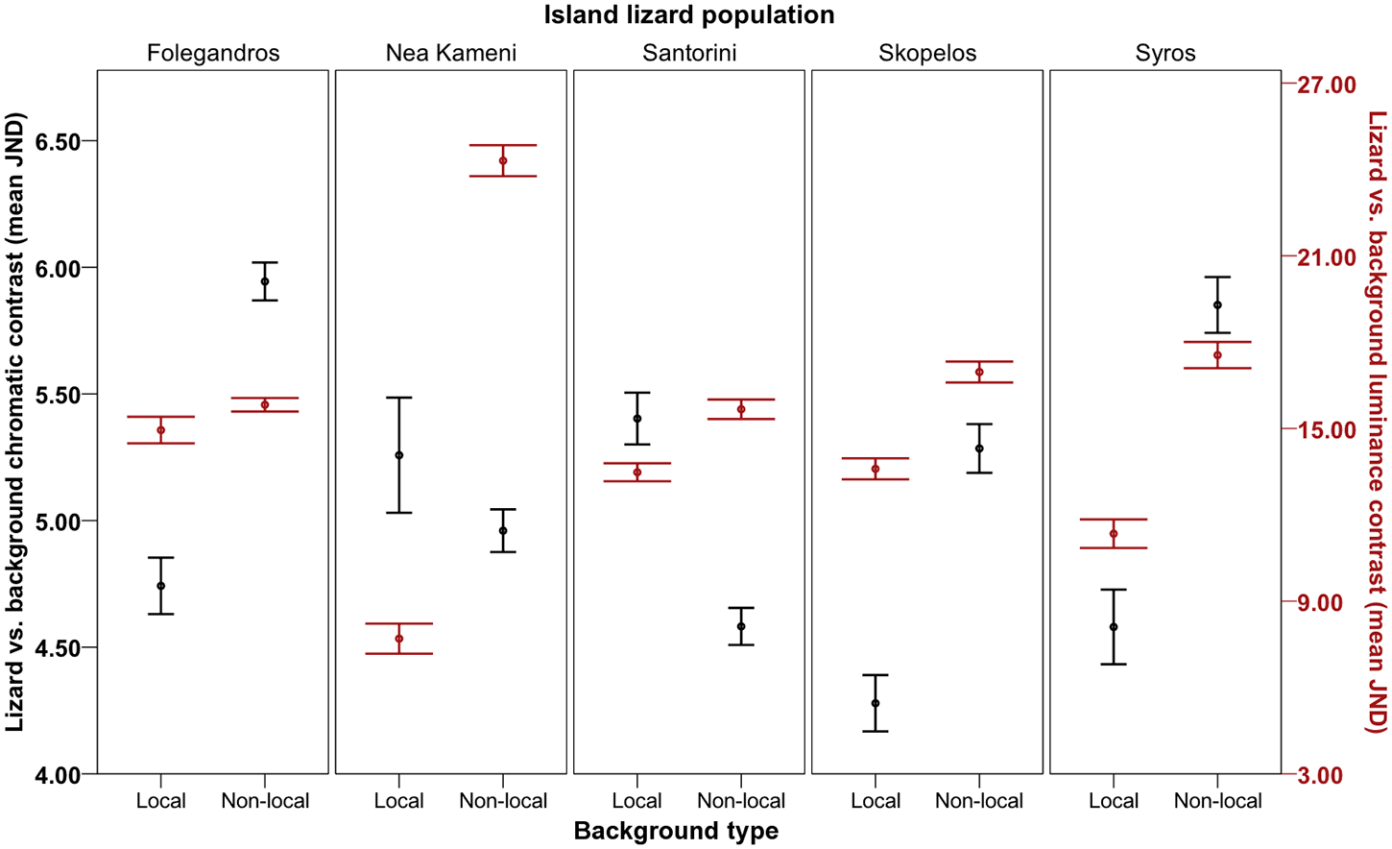
Local vs. non-local camouflage of Aegean wall lizards (*Podarcis erhardii*). Degree of dorsal camouflage is shown against local island backgrounds and against different (non-local) island backgrounds overall (Folegandros, Nea Kameni, Santorini, Skopelos and Syros). Degree of camouflage is shown in terms of chromatic contrast (left axis; black data points) and luminance contrast (right axis; red data points) of lizards’ dorsal regions against the background (mean JND). JND values increasing >3.00 depict lizards that are progressively distinguishable from the background by avian predators. Error bars represent +/- 1 S.E.

Tests 5 and 6 reported a highly significant interaction effect on JNDs (i.e., lizard-background comparisons) between the lizard island populations and the island backgrounds with which they were compared (‘compared island’ background), which explained most of the variance in the achromatic model (Fig 4; Figure A in S3 File; *chromatic*: *F*_10.372, 738.991_ = 5.003, *P* < 0.001, η2 = 0.066; *achromatic*: *F*_6.366, 453.585_ = 30.813, *P* < 0.001, η2 = 0.302). This overall effect varied with sex and body region in both models (Figures B and C in S3 File; *chromatic*: sex, *F*_10.372, 738.991_ = 3.227, *P* < 0.001, η2 = 0.043; body region, *F*_9.954, 709.190_ = 2.358, *P* = 0.010, η2 = 0.032; *achromatic*: sex, *F*_6.366, 453.585_ = 2.992, *P* = 0.006, η2 = 0.040; body region, *F*_9.397, 669.527_ = 3.438, *P* < 0.001, η2 = 0.046). These results revealed that lizards’ dorsal regions were typically better matched against local rock backgrounds than against other (non-local) island rock backgrounds (see Fig 4). However, as predicted, this effect depended on the island population and which other island backgrounds the lizards were compared to, as well as on sex and body region in some populations (Table 2; see Table A and Figures B and C in S3 File).

**Table 2:** Occurrence of enhanced local camouflage in Aegean wall lizards (*Podarcis erhardii*). Enhanced local matching was verified when lizards’ dorsal regions matched their local island backgrounds better than other non-local island backgrounds in terms of chromatic and luminance background matching (JND) to avian predators (see main text and table footnotes for symbol definitions). Lizard and background differences refer to significant differences in degree of contrast (JND) and in colour (*hue* and *saturation*) and *luminance* (see main text) between local and non-local islands.

Local island and lizard subspecies ^†^	Non-local island	Lizard differences? Yes (Y) No (N)	Background differences? Yes (Y) No (N)	Enhanced local camouflage (chromatic)	Enhanced local camouflage (luminance)
**Folegandros** *P*.*e*.*naxensis*	Nea Kameni	Y	Y	**✓**	**✓**
	Santorini	Y	Y	**✓**	**✓♀**
	Skopelos	Y	Y	**✓♂**	**❖**
	Syros	Y	Y	**✓**	**✓**
**Nea Kameni** *P*.*e*.*naxensis*	Folegandros	Y	Y	**❖**	**✓**
	Santorini	Y	Y	**✓**	**✓**
	Skopelos	Y	Y	**❖♀**	**✓**
	Syros	Y	Y	**−**	**✓**
**Santorini** *P*.*e*.*naxensis*	Folegandros	Y	Y	**❖**	**❖♀**
	Nea Kameni	Y	Y	**❖♀**	**✓**
	Skopelos	Y	N (lum) Y (col)	**❖♀**	**✓♂**
	Syros	Y	Y	**❖♂**	**✓♂**
**Skopelos** *P*.*e*.*ruthveni*	Folegandros	Y	Y	**−**	**✓**
	Nea Kameni	Y	Y	**✓**	**✓**
	Santorini	Y	Y	**✓**	**✓**
	Syros	Y	Y	**✓**	**✓**
**Syros** *P*.*e*.*mykonensis*	Folegandros	Y	Y	**−**	**✓♂**
	Nea Kameni	Y	Y	**✓**	**✓**
	Santorini	N (lum) Y (col)	Y	**✓**	**✓**
	Skopelos	Y	Y	**✓**	**✓♂**

**✓** Enhanced camouflage against local backgrounds vs. non-local backgrounds (JND) (*P* < 0.05)

❖ Inferior camouflage against local backgrounds vs. non-local backgrounds (JND) (*P* < 0.05)

**−** No difference in camouflage against local vs. non-local backgrounds (JND) (*P* > 0.05)

**♂**/**♀** Difference in camouflage only in males/females

^†^ Subspecies identified following [23–25].

### Sex differences in local adaptation

Although Folegandros females typically showed better local chromatic camouflage compared to males in some island comparisons (vs. Santorini: F_1, 98_ = 7.602, *P* = 0.007, η2 = 0.072; vs. Syros: F_1, 98_ = 34.316, *P* < 0.001, η2 = 0.259), in other comparisons, better camouflage against local backgrounds was found in only males and in only females (Table 2). Moreover, Santorini and Syros males were sometimes the only sex to show enhanced achromatic local matching (Table 2; Figure C in S3 File).

### Body region differences in local adaptation

As predicted, Folegandros lizards’ lower backs were generally more camouflaged in colour against local backgrounds compared to their upper backs (Figure B in S3 File; vs. Santorini: F_1, 98_ = 18.347, *P* < 0.001, η2 = 0.158; vs. Syros: F_1, 98_ = 5.860, *P* = 0.017, η2 = 0.056; vs. Nea Kameni: F_1, 98_ = 5.414, *P* = 0.022, η2 = 0.052). Similarly, Skopelos lizards’ lower backs sometimes showed relatively better luminance matching against local backgrounds than their upper backs (Figure C in S3 File; vs. Nea Kameni: F_1, 54_ = 5.741, *P* = 0.020, η2 = 0.096). In contrast, in one case, only the upper backs of Folegandros lizards showed enhanced achromatic camouflage against local backgrounds (Table A and Figure C in S3 File; vs Nea Kameni: F_1, 98_ = 17.716, *P* < 0.001, η2 = 0.153).

## Discussion

We investigated whether dorsal coloration among island populations of Aegean wall lizards (*Podarcis erhardii*) differs in order to match local rock backgrounds for camouflage against avian predators. We found that lizards were better camouflaged against local island backgrounds compared to different island backgrounds to reduce detection by avian predators, with few exceptions (see Table 2; Fig 4; Figure A in S3 File). This was typically accompanied by island differences in lizards and the backgrounds they rest on, which were sufficient to be perceived by a typical avian predator visual system (see Table 2; Figs 2 and 3; S1 and S2 Files).

We found that better matching to local backgrounds occurred in most *P*. *erhardii* populations (Fig 4), and this arose when lizards were compared to most other non-local island backgrounds, in terms of luminance and/or chromatic matching (Table 2). These findings suggest that selection has typically driven better matching against local backgrounds to enhance camouflage against avian predators, in line with our predictions and with previous studies on lizards and mice (e.g. [12, 13, 80, 81]). Contrary to our predictions, in most populations, both sexes and dorsal body regions showed equally enhanced camouflage against local backgrounds (with some exceptions; Table 2; Figures B and C in S3 File). This is probably because selection favours camouflage on dorsal regions exposed to aerially hunting birds to offset the relatively heightened conspicuousness in sexually competing males and to further improve female camouflage (e.g. [13, 36, 82]). In addition, we did not find a clear association between predator risk (i.e. number of resident avian predator species; Table 1) and degree of local camouflage. For example, Santorini lizards exhibited relatively poor chromatic camouflage despite being under high risk, while Folegandros lizards were comparatively well camouflaged in their lower risk habitats (see Tables 1 and 2). This unexpected result may be because the number of resident avian predator species does not sufficiently reflect degree of predator pressure on each island. A more rigorous test would need a precise count of resident avian predators (e.g. number of breeding pairs) along with a measurement of relative survival (e.g. survival experiments with artificial models) on each island.

Local achromatic matching was particularly enhanced, with 18 out of 20 island comparisons showing better achromatic matching against local backgrounds (Table 2). This is likely to aid lizard camouflage by preventing discrimination of visual textures and small targets by hunting birds, particularly during motion (escape) ([58, 83, 84]). These results were consistent with frequent differences in lizard luminance (perceived lightness) between islands, and were also substantiated by corresponding patterns of variation between lizards and backgrounds. For example, Nea Kameni lizards and their rock backgrounds were both darker than those of most other islands, and those on Syros were typically lighter (see S1 File). Moreover, in many comparisons, lizards and their backgrounds were more similar in luminance to that of their own population as opposed to non-local populations (Figs 2 and 3; see S2 File). Accordingly, these island populations consistently showed better luminance camouflage to avian predators against local backgrounds than against all non-local backgrounds (Table 2; Fig 4; S3 File). We found only one puzzling result. Syros lizards showed better luminance matching against local backgrounds than against non-local Santorini backgrounds, despite the lizards from these populations being no different in luminance (Table 2). This might be explained by subtle differences in lightness between these populations that were not identified by our analysis, together with the finding that they rest on backgrounds that strongly differ in luminance (see S1 and S2 Files).

Relatively heightened chromatic matching against local backgrounds was found in 11 out of 20 island comparisons (Table 2), which probably reduces the risk of detection by birds ([59, 85]). This consistently occurred when lizards and their backgrounds were more similar in colour to that of their own population than to that of non-local populations (Table 2; S2 File). These results suggest that heightened local chromatic camouflage against avian predators has been caused by adaptive convergence of coloration in each island population to match local backgrounds. Overall, these results suggest that the isolation of certain *P*. *erhardii* island populations in different environments has been generally sufficient to cause local adaptation for camouflage, as indicated by previous work ([36]).

These assumptions are supported by various indications that selection should favour dorsal matching to local backgrounds in *P*. *erhardii*. Past studies have shown that background matching dorsal camouflage in lizards reduces detection by avian predators in local environments (e.g. [12, 13, 36]). In addition, survival experiments with artificial models of *P*. *erhardii* and of other lizards have shown that more conspicuous models are attacked more frequently by avian predators compared to more camouflaged models ([86, 87]). Thus, dorsal matching to local rock backgrounds is likely to counteract considerable risk from avian predators, especially because *P*. *erhardii*, like other lizards, typically bask and display on rocks in open environments where they are highly visible to hunting birds ([31, 37]; pers. observations). Moreover, there is known genetic diversity and little gene flow (via over-water dispersal) between most Aegean island populations ([26, 27, 41]), similarly to other island lizards ([42]). This means that many of the current populations are likely to be descendants of the original colonists, which are highly likely to have adapted to match their native rock backgrounds since their time of isolation. This is supported by previous findings of adaptation to match local backgrounds on a comparably short evolutionary time-scale (i.e., 6,000 years; 10; Table 1). Moreover, our results show better local camouflage relative to non-local camouflage in comparisons between islands that were historically geographically connected and that have the same inhabitant subspecies, again suggesting that isolation of these island populations in their local environment has been sufficient to drive diversification for camouflage (e.g. Folegandros vs. Santorini; [23, 27]; Tables 1 and 2; Figure A in S3 File). However, we cannot rule out the influence of phenotypic plasticity given that (non-rapid) colour change appears to occur in *P*. *erhardii* over different seasonal periods to optimize camouflage (K. Marshall, unpublished data). Indeed, other lizards show similar colour change abilities, such as over different seasonal periods, photoperiods and developmental stages, which have sometimes been shown to enhance camouflage (e.g. [18, 19, 88, 89]).

Although past studies have reported local adaptation of camouflage in sympatric lizard populations (e.g. [10, 12, 13, 80]), here we studied separate island populations with distinct environments and evolutionary histories. This system has been useful in identifying certain factors that may affect local selection for camouflage. In particular, in some island populations, enhanced local background matching (relative to matching against non-local island backgrounds) was not found, or was found only in either chromatic or luminance matching, even when local and non-local lizards and background environments were variable (i.e. in 9 comparisons; Table 2). Often in such cases, the local island had been isolated for a relatively short period of time with potentially high levels of re-colonization from boat traffic (i.e., Nea Kameni; [26, 27]) and had experienced relatively low levels of risk from fewer resident avian predator species (i.e., Folegandros; [31]) (see Tables 1 and 2). In addition, the prolonged (> 200,000 year) period of volcanic eruptions on Santorini and the resulting geological complexity of the island (Table 1; [33, 34]) may have caused the inferior local selection for chromatic camouflage in the inhabitant lizards (Table 2; Fig 4). In particular, volcanic eruptions may have eradicated past populations on Santorini and/or changed the rock backgrounds to which they had become adapted, and produced a more heterogeneous environment to prevent more specialized camouflage ([90, 91]). These findings therefore indicate that, as predicted, local selection for camouflage in certain populations may be influenced to some degree by traits specific to their local island, such as predation risk and biogeographic history. Further survival experiments and genetic analyses would corroborate these ideas.

Nonetheless, enhanced local camouflage was found in relatively young and novel environments with a history of re-colonization, indicating rapid adaptation or phenotypic changes to enhance camouflage. For example, Nea Kameni lizards showed extremely strong achromatic camouflage in the form of dark (almost black) coloration against their black lava backgrounds (Table 2; Fig 4), much like other melanic lizard morphs have adapted to match darker substrates (e.g. [29]). This was despite a potentially high level of gene flow from the nearby Santorini population via unusually large amounts of boat traffic ([26]). The possibility that Nea Kameni lizards have become darker than their recolonizing population to match their darker backgrounds is supported by findings in other island lizards showing that phenotypic divergence has occurred despite high levels of gene flow between populations ([92]). Again, genetic analyses would substantiate these assumptions.

Our key findings show that Aegean wall lizard populations match their local backgrounds better than non-local backgrounds to enhance camouflage against avian predators, similarly to past work (e.g. [12, 13]), and that this appears to depend on ecological conditions and evolutionary history of the local environment. The exact mechanism underlying our results requires corroborative work, including genetic analyses and survival experiments, to investigate the relative roles of genetic adaptation, predation risk and phenotypic plasticity. Additionally, further work exploring how selection may enhance sexual signals against local backgrounds could elucidate the potential for reproductive isolation between the divergent island populations and subsequent speciation.

## Supporting Information

S1 File Island differences in colour and luminance of Aegean wall lizards (*Podarcis erhardii*) and their rock background environments. Occurrence of significant differences and effect sizes (ETA-squared [η^2^]) are shown between island lizard populations (Table A) and between rock backgrounds (Table B) in terms of *hue* (top row), *saturation* (middle) and *luminance* (bottom). Figure A shows variation in dorsal coloration (*hue* and *saturation*) and *luminance* between the island lizard populations (top row) and their rock backgrounds (bottom row).
(DOCX)

S2 File Degree of contrast (JND) between island lizards and between island backgrounds. Occurrence of significant differences and effect sizes (ETA-squared [η^2^]) are shown from statistical analyses comparing degree of contrast (JND) between island populations of Aegean wall lizards (*Podarcis erhardii*) and degree of contrast between their rock backgrounds (Table A). Chromatic and luminance JNDs are shown between island lizard populations (Figure A) and between their rock backgrounds (Figure B).
(DOCX)

S3 File Island differences in local camouflage. Showing significant differences and effect sizes (ETA-squared [η^2^]) comparing degree of local camouflage between different island populations of Aegean wall lizards (*Podarcis erhardii*) (Table A). Degree of dorsal chromatic and luminance camouflage against local and non-local island backgrounds is shown in Figure A. Body region and sex differences in degree of chromatic camouflage (Figure B) and luminance camouflage (Figure C) are shown against local and non-local island backgrounds.
(DOCX)
