# Supplementary material for: Intraspecific Colour Variation among Lizards in Distinct Island Environments Enhances Local Camouflage
Source: PLoS One. 2015 Sep 15;10(9):e0135241. doi: 10.1371/journal.pone.0135241 (PMC4570707; doi:10.1371/journal.pone.0135241)
Supplement: S1 File — Occurrence of significant differences and effect sizes (ETA-squared [η2]) are shown between island lizard populations (Table A) and between rock backgrounds (Table B) in terms of hue (top row), saturation (middle) and luminance (bottom). Figure A shows variation in dorsal coloration (hue and saturation) and luminance between the island lizard populations (top row) and their rock backgrounds (bottom row). (DOCX) [file pone.0135241.s001.docx]

**Table A. Differences in dorsal coloration and luminance between island populations of Aegean wall lizards (*Podarcis erhardii*).** Top: Hue is based on a ratio of avian cone catches (see main text). Middle: Saturation is the distance from the grey point in tetrahedral space. Bottom: Luminance (perceived lightness) is derived from avian double cone catches. Effect sizes (ETA-squared [η^2^]) are shown. See footnotes for symbol definitions.

| **Island population**  ***subspecies***^†^ | **Lizard chromatic differences (hue)** | | | | | | | | | | | | |
| --- | --- | --- | --- | --- | --- | --- | --- | --- | --- | --- | --- | --- | --- |
|  | Folegandros | | Nea Kameni | | | Santorini | | | Skopelos | | | Syros | |
| Folegandros  *P.e.naxensis* |  | | ✔♂  0.104 | | | **−** | | | **−** | | | ✔♀LB  0.066 | |
| Nea Kameni  *P.e.naxensis* | ✔♂  0.104 | |  | | | ✔♂UB  0.113 | | | **−** | | | ✔  0.059 | |
| Santorini  *P.e.naxensis* | **−** | | ✔♂UB  0.113 | | |  | | | **−** | | | **−** | |
| Skopelos  *P.e.ruthveni* | **−** | | **−** | | | **−** | | |  | | | **−** | |
| Syros  *P.e.mykonensis* | ✔♀LB  0.066 | | ✔  0.059 | | | **−** | | | **−** | | |  | |
| **Island population**  ***subspecies***^†^ | **Lizard chromatic differences (saturation)** | | | | | | | | | | | | |
|  | Folegandros | | | Nea Kameni | | | Santorini | | | Skopelos | | | Syros |
| Folegandros  *P.e.naxensis* |  | **−** | | | **−** | | | **−** | | | ✔♂UB  0.079 | | |
| Nea Kameni  *P.e.naxensis* | **−** |  | | | ✔♀UB  0.051 | | | **−** | | | ✔UB  0.072 | | |
| Santorini  *P.e.naxensis* | **−** | ✔♀UB  0.051 | | |  | | | **−** | | | **−** | | |
| Skopelos  *P.e.ruthveni* | **−** | **−** | | | **−** | | |  | | | **−** | | |
| Syros  *P.e.mykonensis* | ✔♂UB  0.079 | ✔UB  0.072 | | | **−** | | | **−** | | |  | | |
| **Island population**  ***subspecies***^†^ | **Lizard achromatic differences (luminance)** | | | | | | | | | | | | |
|  | Folegandros | | Nea Kameni | | | Santorini | | | Skopelos | | | Syros | |
| Folegandros  *P.e.naxensis* |  | | ✔  0.254 | | | ✔♀  0.150 | | | ✔♂UB♀LB  0.068, 0.048 | | | ✔  0.150 | |
| Nea Kameni  *P.e.naxensis* | ✔  0.254 | |  | | | ✔  0.496 | | | ✔  0.271 | | | ✔  0.580 | |
| Santorini  *P.e.naxensis* | ✔♀  0.150 | | ✔  0.496 | | |  | | | ✔♂  0.153 | | | **−** | |
| Skopelos  *P.e.ruthveni* | ✔♂UB♀LB  0.068, 0.058 | | ✔  0.271 | | | ✔♂  0.153 | | |  | | | ✔  0.156 | |
| Syros  *P.e.mykonensis* | ✔  0.150 | | ✔  0.580 | | | **−** | | | ✔  0.156 | | |  | |

✔ Statistically significant difference between island lizard populations (*P* < 0.05)

**−** No statistically significant difference between island lizard populations (*P* > 0.05)

UB/LB: difference only in upper backs/lower backs

♀/♂: difference only in females/males

^†^ Subspecies of each island population identified following (1-3).

**Table B: Differences in background rock colour (hue and saturation) and luminance between the island environments of different populations of Aegean wall lizards (*Podarcis erhardii*).** Top: Hue (colour type) values are based on a ratio of avian predator cone catches (see main text). Middle: Saturation is the distance from the achromatic grey point in tetrahedral colour space of an avian predator. Bottom: Luminance (perceived lightness) was derived from avian predator double cone catches. Effect sizes (ETA-squared [η^2^]) are shown. See footnotes for definition of symbols.

| **Island** | **Background chromatic differences (hue)** | | | | | | | | | |
| --- | --- | --- | --- | --- | --- | --- | --- | --- | --- | --- |
|  | Folegandros | | | Nea Kameni | | Santorini | | Skopelos | | Syros |
| Folegandros |  | | ✔  0.103 | | ✔  0.025 | | **−** | | **−** | |
| Nea Kameni | ✔  0.103 | |  | | **−** | | ✔  0.125 | | ✔  0.090 | |
| Santorini | ✔  0.025 | | **−** | |  | | **−** | | **−** | |
| Skopelos | **−** | | ✔  0.125 | | **−** | |  | | **−** | |
| Syros | **−** | | ✔  0.090 | | **−** | | **−** | |  | |
| **Island** | **Background chromatic differences (saturation)** | | | | | | | | | |
|  | Folegandros | | | Nea Kameni | | Santorini | | Skopelos | | Syros |
| Folegandros |  | | ✔  0.267 | | ✔  0.177 | | ✔  0.028 | | **−** | |
| Nea Kameni | ✔  0.267 | |  | | **−** | | ✔  0.175 | | ✔  0.235 | |
| Santorini | ✔  0.177 | | **−** | |  | | ✔  0.085 | | ✔  0.111 | |
| Skopelos | ✔  0.028 | | ✔  0.175 | | ✔  0.085 | |  | | **−** | |
| Syros | **−** | | ✔  0.235 | | ✔  0.111 | | **−** | |  | |
| **Island** | **Background achromatic differences (luminance)** | | | | | | | | | |
|  | Folegandros | Nea Kameni | | | | Santorini | | Skopelos | | Syros |
| Folegandros |  | | ✔  0.546 | | ✔  0.037 | | ✔  0.110 | | **−** | |
| Nea Kameni | ✔  0.546 | |  | | ✔  0.385 | | ✔  0.355 | | ✔  0.659 | |
| Santorini | ✔  0.037 | | ✔  0.385 | |  | | **−** | | ✔  0.086 | |
| Skopelos | ✔  0.110 | | ✔  0.355 | | **−** | |  | | ✔  0.188 | |
| Syros | **−** | | ✔  0.659 | | ✔  0.086 | | ✔  0.188 | |  | |

✔ Statistically significant difference between island rock backgrounds (*P* < 0.05)

**−** No difference between island rock backgrounds (*P* > 0.05)

**i) Lizard differences**

**ii) Background differences**

**Figure A. Island differences in colour and luminance of Aegean wall lizards (*Podarcis erhardii*) and rock background environments. Top (i):** variation in dorsal coloration (hue and saturation) and luminance between five island populations of *P. erhardii* (Folegandros, Nea Kameni, Santorini, Skopelos and Syros). **Bottom (ii):** differences in coloration and luminance of lizards’ local rock backgrounds between the five islands. Hue values are based on a ratio of avian predator cone catches and saturation values are the distance from the achromatic grey point in tetrahedral colour space of an avian predator (see main text), with larger values representing colours that are more saturated (between 0 and 0.75). Luminance values represent avian predator double cone catches (between 0 and 1). Error bars represent ±1.00 S.E.

**References**

1. Chondropoulos, BP. A checklist of the Greek reptiles. I. The lizards. Amphibia-Reptilia*.* 1986*;* 7: 217-235.

2. Poulakakis N, Lymberakis P, Antoniou A, Chalkia D, Zouros E, Mylonas M, et al. Molecular phylogeny and biogeography of the wall-lizard *Podarcis erhardii* (Squamata: Lacertidae). Mol Phylogenet Evol. 2003; 28(1): 38-46.

3. Poulakakis, N, Goulielmos, G, Antoniou, A, Zouros, E, & Mylonas, M. Isolation and characterization of polymorphic microsatellite markers in the wall lizard *Podarcis erhardii* (Squamata: Lacertidae). Mol. Ecol. Notes. 2005; 5: 549-551.
