# Supplementary material for: Intraspecific Colour Variation among Lizards in Distinct Island Environments Enhances Local Camouflage
Source: PLoS One. 2015 Sep 15;10(9):e0135241. doi: 10.1371/journal.pone.0135241 (PMC4570707; doi:10.1371/journal.pone.0135241)
Supplement: S2 File — Occurrence of significant differences and effect sizes (ETA-squared [η2]) are shown from statistical analyses comparing degree of contrast (JND) between island populations of Aegean wall lizards (Podarcis erhardii) and degree of contrast between their rock backgrounds (Table A). Chromatic and luminance JNDs are shown between island lizard populations (Figure A) and between their rock backgrounds (Figure B). (DOCX) [file pone.0135241.s002.docx]

**Table A: Degree of contrast between island lizards and between island backgrounds.** Showing effect sizes (ETA-squared [η^2^]) from statistical analyses comparing degree of contrast (JND) between island populations of Aegean wall lizards (*Podarcis erhardii*) and degree of contrast between their rock backgrounds. This determined whether lizards and backgrounds they were more similar to those of the local island as opposed to the non-local island, in terms of colour (col) and luminance (lum) to avian predators (increased local similarity; see footnotes).

| **Local island and lizard subspecies** ^†^ | **Non-local island** | **Increased local similarity in lizards** | | **Increased local similarity in backgrounds** | |
| --- | --- | --- | --- | --- | --- |
|  |  | **Col** | **Lum** | **Col** | **Lum** |
| **Folegandros**  *P.e.naxensis* | Nea Kameni | ✔ 0.286 | ✔ 0.181 | ✔ 0.324 | ✔ 0.612 |
|  | Santorini | ✔**♂** 0.074 | ✔**♀** 0.309 | ✔ 0.662 | ✔ 0.475 |
|  | Skopelos | ✔ 0.468 | ✔ 0.359 | ✔ 0.324 | ✔ 0.284 |
|  | Syros | ✔**♀** 0.283 | ✔ 0.344 | ✔ 0.197 | ✔ 0.055 |
| **Nea Kameni**  *P.e.naxensis* | Folegandros | ✔ 0.181 | ✔**♂** 0.491 | ✔ 0.471 | ✔ 0.723 |
|  | Santorini | **−** | ✔ 0.834 | ✔ 0.570 | ✔ 0.735 |
|  | Skopelos | ✔ 0.240 | ✔ 0.703 | ✔ 0.554 | ✔ 0.639 |
|  | Syros | ✔ 0.414 | ✔ 0.838 | ✔ 0.526 | ✔ 0.804 |
| **Santorini**  *P.e.naxensis* | Folegandros | ✔ 0.429 | **−** | ❖0.232 | ❖0.320 |
|  | Nea Kameni | ✔ 0.312 | ✔ 0.416 | ❖0.419 | ✔ 0.125 |
|  | Skopelos | ✔ 0.409 | ✔UB 0.254 | **−** | **−** |
|  | Syros | ✔**♀** 0.339 | ✔ 0.149 | **−** | **−** |
| **Skopelos**  *P.e.ruthveni* | Folegandros | ✔ 0.481 | ❖0.197 | **−** | **−** |
|  | Nea Kameni | ✔ 0.392 | **−** | ✔ 0.348 | ✔ 0.198 |
|  | Santorini | ✔**♂** UB 0.182 | **−** | ✔ 0.406 | ✔ 0.108 |
|  | Syros | ✔ 0.379 | ✔**♂** 0.103 | ✔ 0.084 | **−** |
| **Syros**  *P.e.mykonensis* | Folegandros | ✔UB 0.384 | ✔**♂** 0.105 | **−** | ✔ 0.134 |
|  | Nea Kameni | ✔**♂** UB 0.253 | ✔ 0.335 | **−** | ✔ 0.539 |
|  | Santorini | **−** | ✔**♂** 0.082 | ✔ 0.557 | ✔ 0.456 |
|  | Skopelos | ✔**♂** 0.184 | ✔**♂** 0.143 | ✔ 0.166 | ✔ 0.330 |

✔ Increased local similarity in lizards/backgrounds (vs. non-local lizards/backgrounds; *P* < 0.05)

❖ Decreased local similarity in lizards/backgrounds (vs. non-local lizards/backgrounds; *P* < 0.05)

**−** No difference in degree of contrast between local vs. non-local lizards/backgrounds (*P* > 0.05)

**♂**/**♀** - Difference in degree of contract only in male/female lizards

UB – Difference in degree of contrast only in lizard upper backs

^†^ Subspecies of each island population identified following (1-3).


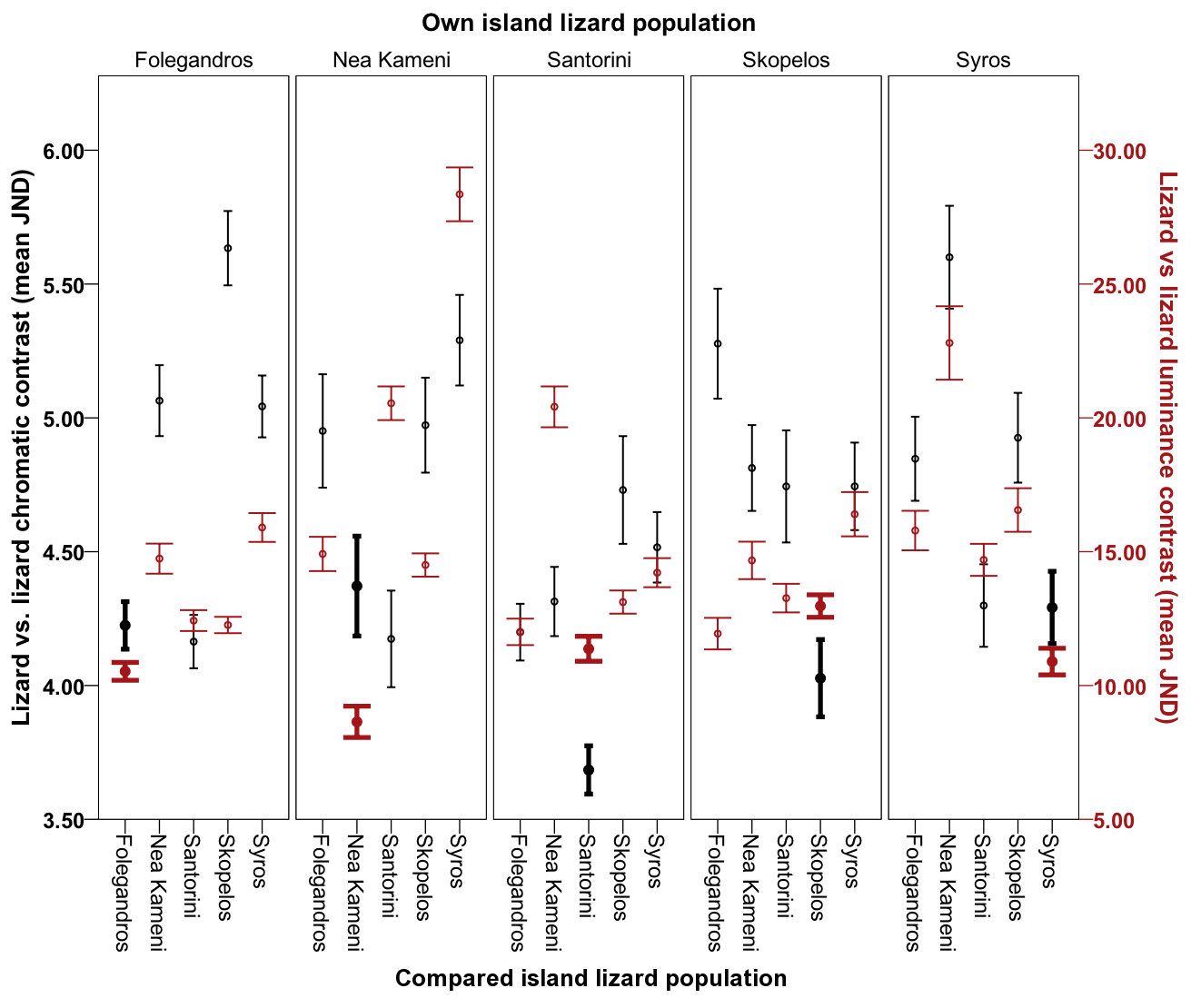


**Figure A.** **Differences in degree of contrast (JND) between island populations of Aegean wall lizards (*Podarcis erhardii*).** Showing how different lizards are to their local island population (shown in bold) and how different they are to each of the other (non-local) island populations (Folegandros, Nea Kameni, Santorini, Skopelos and Syros). Differences are shown in terms of chromatic contrast (left axis; black data points) and luminance contrast (right axis; red data points) (mean JND). JND values increasing >3.00 depict populations that are progressively distinguishable by avian predators. Error bars represent +/- 1 S.E.


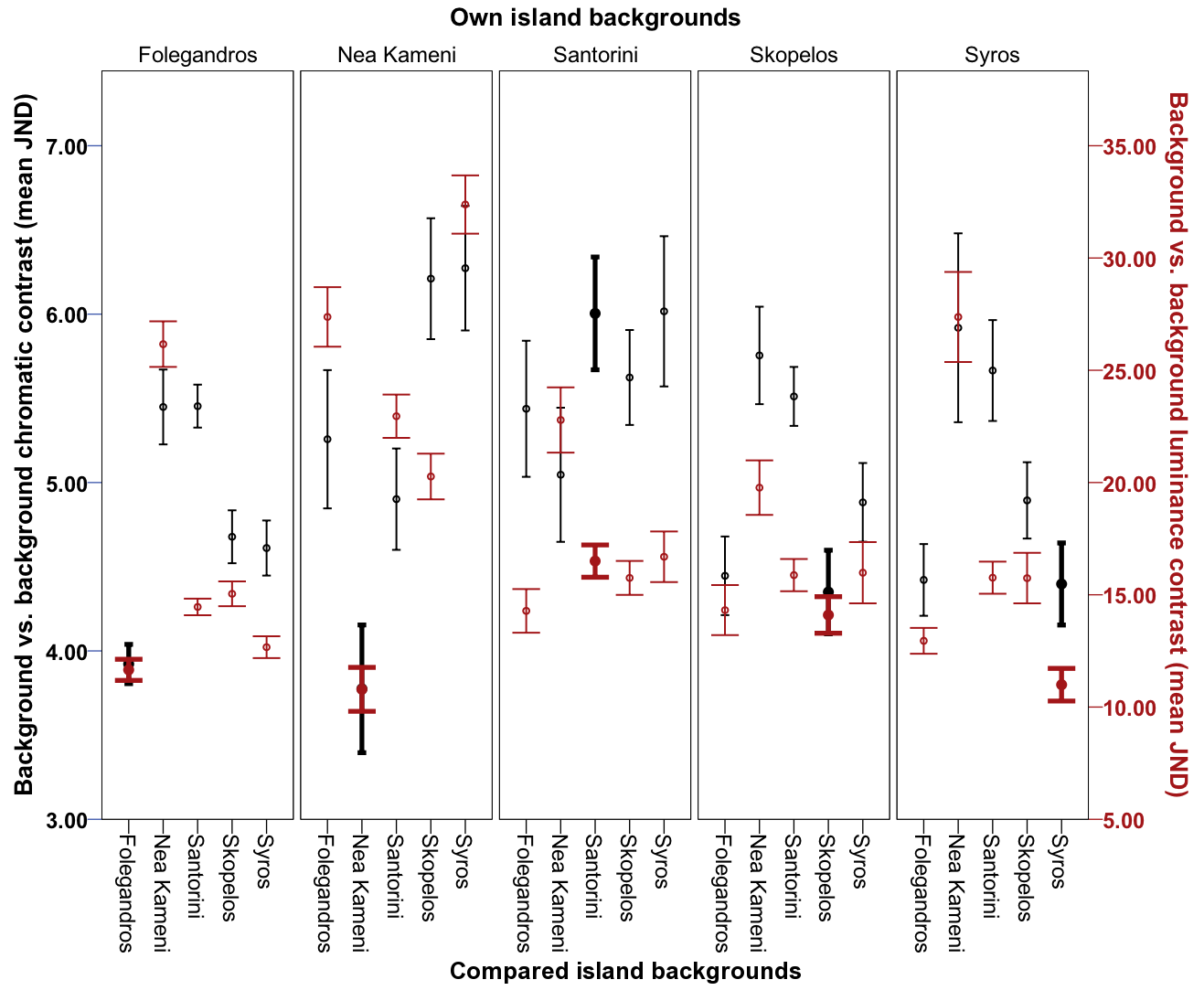


**Figure B.** **Differences in degree of contrast (JND) between island rock backgrounds of Aegean wall lizards (*Podarcis erhardii*).** Showing how different rock backgrounds are to those of their local island (shown in bold) and how different they are to backgrounds of the other (non-local) island populations (Folegandros, Nea Kameni, Santorini, Skopelos and Syros). Differences are shown in terms of chromatic contrast (left axis; black data points) and luminance contrast (right axis; red data points) (mean JND). JND values increasing >3.00 depict populations that are progressively distinguishable by avian predators. Error bars represent +/- 1 S.E.

**References**

1. Chondropoulos, BP. A checklist of the Greek reptiles. I. The lizards. Amphibia-Reptilia*.* 1986*;* 7: 217-235.

2. Poulakakis N, Lymberakis P, Antoniou A, Chalkia D, Zouros E, Mylonas M, et al. Molecular phylogeny and biogeography of the wall-lizard *Podarcis erhardii* (Squamata: Lacertidae). Mol Phylogenet Evol. 2003; 28(1): 38-46.

3. Poulakakis, N, Goulielmos, G, Antoniou, A, Zouros, E, & Mylonas, M. Isolation and characterization of polymorphic microsatellite markers in the wall lizard *Podarcis erhardii* (Squamata: Lacertidae). Mol. Ecol. Notes. 2005; 5: 549-551.
