# Supplementary material for: Intraspecific Colour Variation among Lizards in Distinct Island Environments Enhances Local Camouflage
Source: PLoS One. 2015 Sep 15;10(9):e0135241. doi: 10.1371/journal.pone.0135241 (PMC4570707; doi:10.1371/journal.pone.0135241)
Supplement: S3 File — Showing significant differences and effect sizes (ETA-squared [η2]) comparing degree of local camouflage between different island populations of Aegean wall lizards (Podarcis erhardii) (Table A). Degree of dorsal chromatic and luminance camouflage against local and non-local island backgrounds is shown in Figure A. Body region and sex differences in degree of chromatic camouflage (Figure B) and luminance camouflage (Figure C) are shown against local and non-local island backgrounds. (DOCX) [file pone.0135241.s003.docx]

**Table A: Local adaptation of camouflage.** Showing effect sizes (ETA-squared [η^2^]) from statistical analyses comparing degree of local camouflage between different island populations of Aegean wall lizards (*Podarcis erhardii*). Lizards were compared with local and non-local backgrounds (JND) to test whether they matched local backgrounds better for camouflage, in terms of colour and luminance to avian predators (enhanced local camouflage; see footnotes).

| **Local island and lizard subspecies** ^†^ | **Other island backgrounds** | **Enhanced local camouflage - chromatic** | **Enhanced local camouflage - luminance** |
| --- | --- | --- | --- |
| **Folegandros**  *P.e.naxensis* | Nea Kameni | ✔ 0.534 | ✔UB 0.153 |
|  | Santorini | ✔ 0.790 | ✔**♀** 0.115 |
|  | Skopelos | ✔**♂** 0.202 | ❖ 0.107 |
|  | Syros | ✔ 0.393 | ✔ 0.211 |
| **Nea Kameni**  *P.e.naxensis* | Folegandros | ❖ 0.175 | ✔ 0.803 |
|  | Santorini | ✔ 0.247 | ✔ 0.834 |
|  | Skopelos | ❖**♀** 0.158 | ✔ 0.707 |
|  | Syros | − | ✔ 0.766 |
| **Santorini**  *P.e.naxensis* | Folegandros | ❖ 0.753 | ❖**♀** 0.111 |
|  | Nea Kameni | ❖**♀** 0.120 | ✔ 0.076 |
|  | Skopelos | ❖**♀** 0.326 | ✔**♂** 0.248 |
|  | Syros | ❖**♂** 0.160 | ✔**♂** 0.206 |
| **Skopelos**  *P.e.ruthveni* | Folegandros | − | ✔ 0.074 |
|  | Nea Kameni | ✔ 0.379 | ✔ 0.127 |
|  | Santorini | ✔ 0.494 | ✔ 0.238 |
|  | Syros | ✔ 0.075 | ✔ 0.254 |
| **Syros**  *P.e.mykonensis* | Folegandros | − | ✔**♂** 0.289 |
|  | Nea Kameni | ✔ 0.600 | ✔ 0.574 |
|  | Santorini | ✔ 0.711 | ✔ 0.437 |
|  | Skopelos | ✔ 0.151 | ✔**♂** 0.245 |

✔ Better camouflage against local island backgrounds (vs. non-local island backgrounds; *P* < 0.05)

❖ Inferior camouflage against local island backgrounds (vs. non-local island backgrounds; *P* < 0.05)

**−** No difference in camouflage between comparisons with local vs. non-local backgrounds; *P* > 0.05)

**♂**/**♀** - Better local camouflage only in males/females

UB - Better local camouflage only in upper backs

^†^ Subspecies of each island population identified following (1-3).


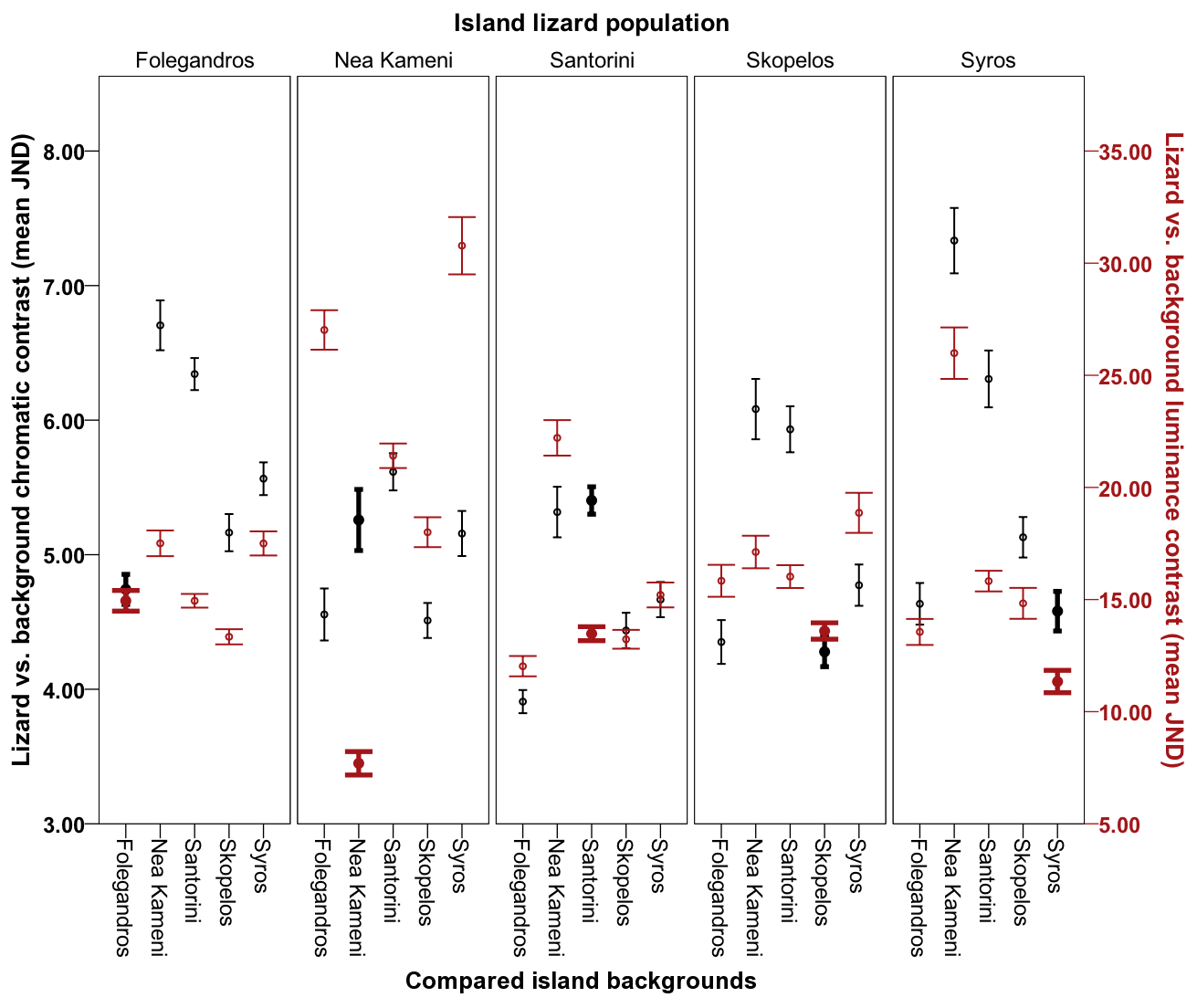


**Figure A:** **Island differences in local camouflage.** Showing degree of dorsal camouflage of Aegean wall lizards (*Podarcis erhardii*) against local island backgrounds (shown in bold) and against each of the different (non-local) island backgrounds (Folegandros, Nea Kameni, Santorini, Skopelos and Syros). Degree of camouflage is shown in terms of chromatic contrast (left axis; black data points) and luminance contrast (right axis; red data points) of lizards’ dorsal regions against the background (mean JND). JND values increasing >3.00 depict lizards that are progressively distinguishable from the background by avian predators. Error bars represent +/- 1 S.E.

**
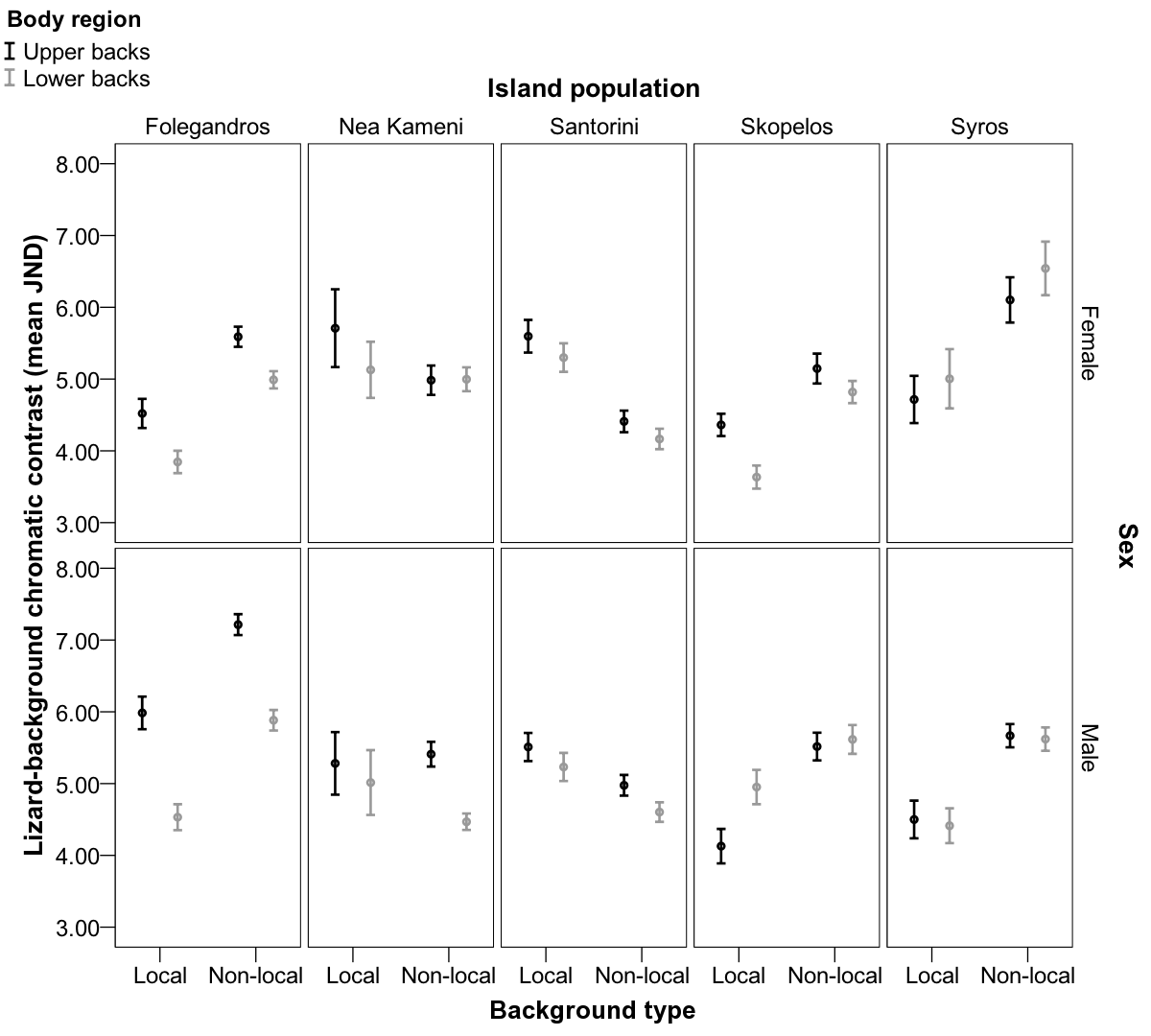
**

**Figure B. Body region and sex differences in local chromatic camouflage.** Showing differences between sexes and upper and lower backs in the degree of chromatic background matching camouflage of Aegean wall lizards (*Podarcis erhardii*) against local island backgrounds and against different (non-local) island backgrounds (Folegandros, Syros, Santorini, Nea Kameni and Skopelos). Error bars represent +/- 1 S.E.


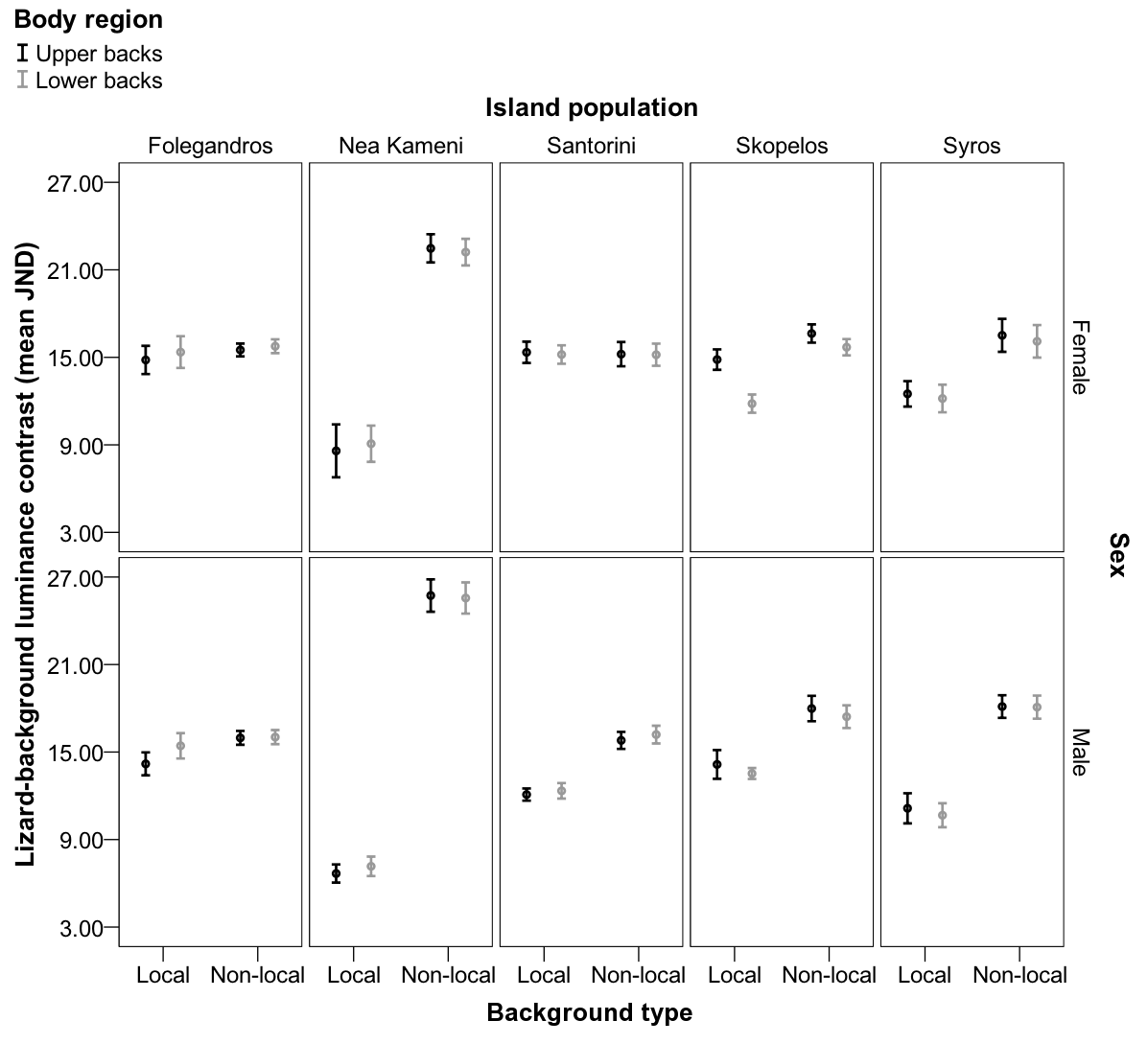


**Figure C. Body region and sex differences in local luminance camouflage.** Showing differences between sexes and upper and lower backs in the degree of luminance background matching camouflage of Aegean wall lizards (*Podarcis erhardii*) against local island backgrounds and against different (non-local) island backgrounds (Folegandros, Syros, Santorini, Nea Kameni and Skopelos). Error bars represent +/- 1 S.E.
